# Combining Google Earth historical imagery and UAV photogrammetry for urban development analysis

**DOI:** 10.1016/j.mex.2024.102785

**Published:** 2024-06-03

**Authors:** Chima Iheaturu, Chukwuma Okolie, Emmanuel Ayodele, Andy Egogo-Stanley, Solomon Musa, Chinwe Ifejika Speranza

**Affiliations:** a University of Bern, Institute of Geography, Bern, Switzerland; b University of Lagos, Department of Surveying and Geoinformatics, Lagos, Nigeria; c Federal Capital Development Authority, Department of Survey and Mapping, Abuja, Nigeria

**Keywords:** Google Earth Historical Imagery and Structure-from-Motion photogrammetry-based urban development monitoring approach, Structure-from-motion photogrammetry, Change detection analysis, Parsimonious land use, Building heights, digital map creation

## Abstract

Rural-urban migration often triggers additional demand for housing and infrastructural development to cater for the growing population in urban areas. Consequently, town planners and urban development authorities need to understand the urban development trend to make sustainable urban planning decisions. Yet, methods to analyse changes and trends in urban spatial development are often complex and require costly data collection. This article thus presents a simplified method to analyse the urban development trend in an area. The method integrates Google Earth (GE) historical imagery (baseline data) and unmanned aerial vehicle (UAV) photogrammetry (recent data) to quantify the changes over time. This approach can be applied to study the urban development trends in low-income countries with budget constraints. The method is discussed under four main headings: (1) background, (2) method details, (3) limitations, and (4) conclusion.

• Google Earth historical image can be extracted with its associated world file.
• The population of an area can be estimated by using average household size data and the number of residential buildings in the area.
• The building height ratio can be used to ascertain if the land is being used parsimoniously.

## Graphical abstract

**Figure:**
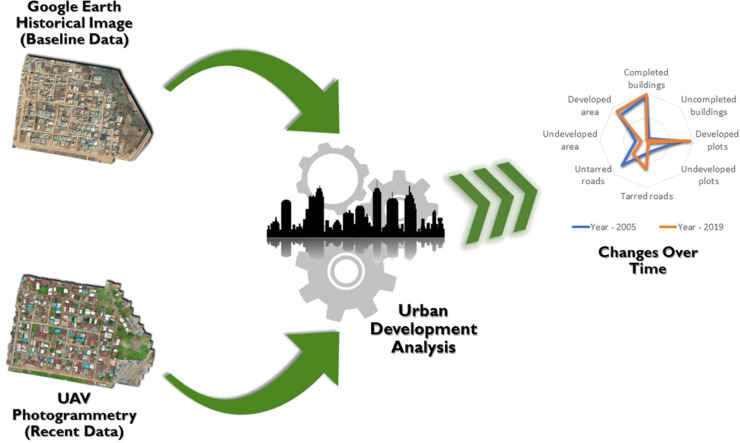

Specifications table

**Table:** 

Subject area:	Environmental Sciences
More specific subject area:	Urban development analysis
Name of your method:	Google Earth Historical Imagery and Structure-from-Motion photogrammetry-based urban development monitoring approach
Name and reference of original method:	Iheaturu, C., Okolie, C., Ayodele, E., Egogo-Stanley, A., Musa, S., & Ifejika Speranza, C. (2022). A simplified structure-from-motion photogrammetry approach for urban development analysis. Remote Sensing Applications: Society and Environment, 28. https://doi.org/10.1016/j.rsase.2022.100850 Manesha, E. P. P., Jayasinghe, A., & Kalpana, H. N. (2021). Measuring urban sprawl of small and medium towns using GIS and remote sensing techniques: A case study of Sri Lanka. *Egyptian Journal of Remote Sensing and Space Science, 24*(3P2). https://doi.org/10.1016/j.ejrs.2021.11.001 Wang, H., Gong, X., Wang, B., Deng, C., & Cao, Q. (2021). Urban development analysis using built-up area maps based on multiple high-resolution satellite data. *International Journal of Applied Earth Observation and Geoinformation, 103*. https://doi.org/10.1016/j.jag.2021.102500
Resource availability:	Not applicable

## Background

Urbanisation is a global phenomenon rapidly transforming landscapes across the world. The United Nations projects that by 2050, 68 % of the global population will reside in urban areas, a significant increase from the current 55 % [1]. This massive shift necessitates accurate and timely spatial data to support urban planning and management. However, in many developing regions, access to high-resolution spatial data and advanced remote sensing technologies is often limited due to financial constraints, technical expertise shortages, and infrastructural challenges. This limitation hinders the ability to effectively monitor and analyse urban growth patterns, which are crucial for informed urban planning, decision-making, and sustainable development. This is particularly significant for rapidly developing regions, such as the Kuje Area Council in Nigeria's Federal Capital Territory (FCT).

To address these challenges, this study presents a detailed methodology for generating high-resolution spatial data products for comprehensive urban development analysis in Kuje, using an integrated approach. This region is experiencing rapid population growth and urban expansion, which, without proper monitoring and management, can lead to issues such as environmental degradation, overburdened infrastructure, and increased social inequalities. The presented methodology leverages freely available Google Earth historical imagery as a baseline and then combines it with recent Structure-from-Motion (SfM) photogrammetry-derived imagery. The goal is to produce accurate and up-to-date spatial data products in a manner that is both accessible and reproducible, even in areas with limited resources or access to advanced remote sensing data.

The motivation for developing and providing this methodology stems from the need to facilitate broader adoption and implementation of innovative approaches to urban development analysis, especially in data-scarce regions. The original research by Iheaturu et al. [2] introduced a simplified SfM photogrammetry approach for analyzing urban developments in a suburban area of Abuja, Nigeria. However, the study did not encompass an assessment of urban development intensity, which is crucial for understanding the speed, magnitude, and sustainability of urban growth [[3], [4], [5]].

This paper extends the original work by incorporating metrics for urban development intensity and parsimonious land use, thereby providing deeper insights into urban growth patterns. These metrics help identify areas where vertical growth can be encouraged to optimize land use and mitigate urban sprawl. By offering a comprehensive and accessible description of this integrated approach, this methodology aims to empower researchers and practitioners in data-scarce regions with the necessary guidance on data acquisition, processing, and analysis techniques. Ultimately, this paper contributes to the expanding body of literature on the applications of SfM in urban studies while enhancing the reproducibility and transparency of the original research. By doing so, it supports informed urban planning and management decisions that promote sustainable and equitable urban development. The methodology described herein not only builds on the foundational work of Iheaturu et al. [2] but also provides a practical and scalable approach that can be widely adopted to address the pressing needs of urbanization in various contexts.

## Method details

Understanding the urban growth dynamics in Kuje is essential for developing strategies to mitigate these negative impacts and promote sustainable development. In this context, innovative and cost-effective approaches that leverage readily available data sources and cutting-edge technologies are imperative. The methodology presented in this study addresses this need by integrating freely available historical imagery with recent data derived from Structure-from-Motion (SfM) photogrammetry, a technique that can produce high-resolution spatial data. This approach is designed to be accessible and reproducible, even in resource-constrained settings, making it a valuable tool for urban development analysis in such regions.

**Fig. 1:**
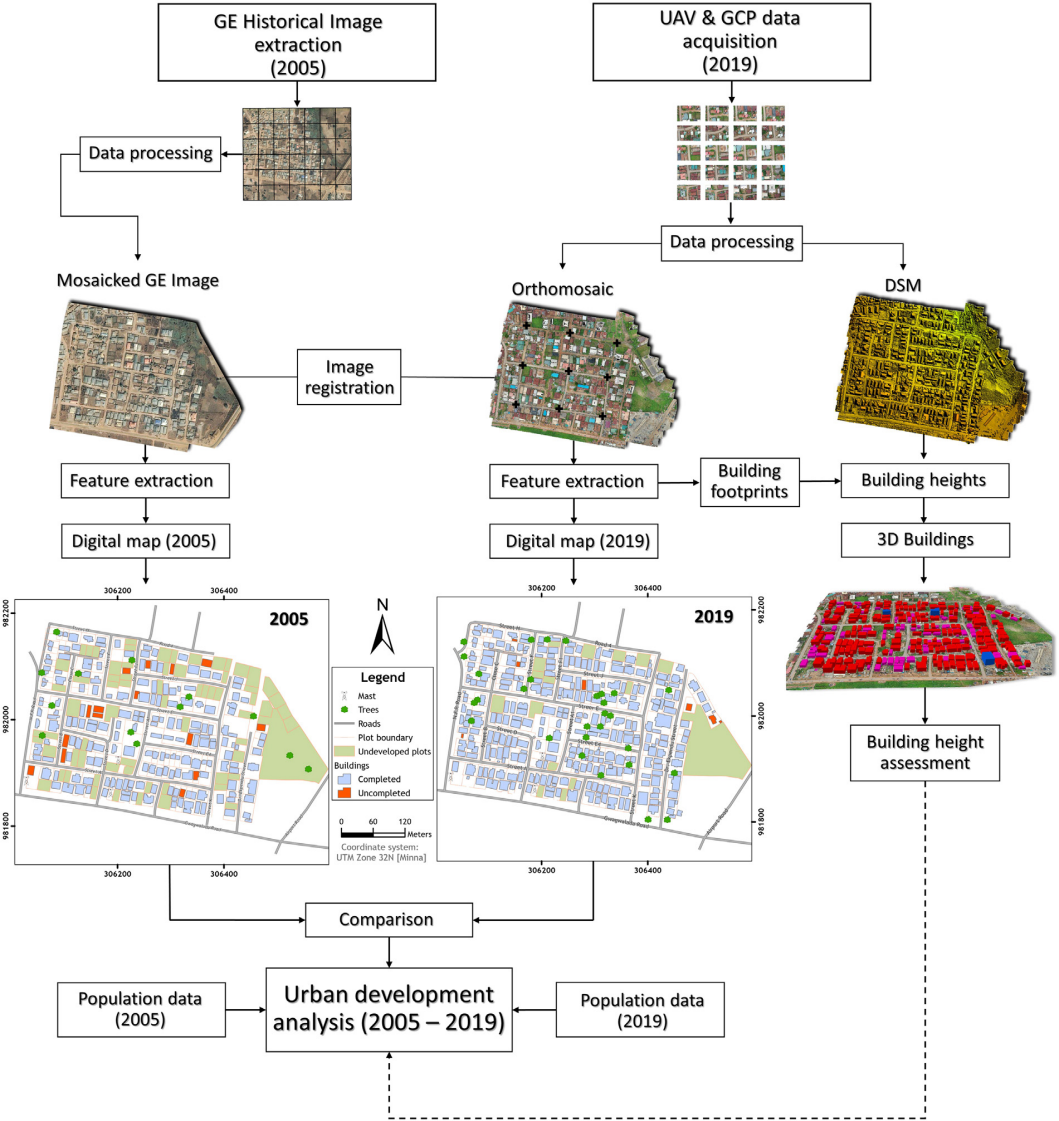
Schematic flowchart of the main steps for urban development analysis of a section of Kuje Area Council. The visualisations presented in this figure were adapted from Iheaturu et al. [2].

The method developed in this study is crucial for several reasons:

*Accessibility:* By using freely available historical imagery from Google Earth and combining it with recent SfM photogrammetry data, this method overcomes the financial and technical barriers that often restrict access to high-resolution spatial data in developing regions.
*Cost-Effectiveness:* The reliance on accessible data sources and simplified photogrammetry techniques reduces the cost associated with acquiring and processing high-resolution spatial data, making it feasible for regions with limited resources.
*Enhanced monitoring:* The integrated approach provides detailed and accurate spatial data products, enabling comprehensive monitoring of urban growth patterns. This is essential for informed urban planning and sustainable development, particularly in rapidly urbanizing areas like Kuje.
*Reproducibility:* The methodology is designed to be easily replicable, allowing other researchers and practitioners in similar contexts to adopt and implement the approach. This promotes wider adoption and contributes to a more extensive understanding of urban growth dynamics across different regions.
*Support for sustainable development:* By providing detailed insights into urban growth patterns, the methodology supports the development of strategies to manage urban expansion sustainably. This can help mitigate negative impacts such as environmental degradation, infrastructure strain, and social inequalities. Fig. 1 presents a methodological flow chart summarising the main steps of the simplified urban development analysis workflow.

## Data acquisition and processing

### GE historical imagery

We utilised a high-resolution (14 cm) Google Earth (GE) historical image of 2005 to serve as the baseline data to quantify the urban development in a section of Kuje Area Council in Abuja, Nigeria. The high-resolution GE images were extracted using the open-source software Elshayal Smart GIS [6]. Elshayal software links with GE Pro-and downloads the satellite image on display in GE Pro. To ensure that an optimal resolution is achieved throughout, it is important to create an index grid with dimensions (height and width) of the best possible zoom level (screen) for the area. This zoom level (screen) dimension can be determined by simultaneously adjusting the zoom slider and visualising the screen until the image is clear enough and then the height and width of the screen are measured. The resultant index grid for this study contained a total of 42 square polygons measuring 100 m by 100 m. This index grid ensured that the images were downloaded systematically at a uniform resolution. Since our interest was to acquire the 2005 imagery, we used the Historical Imagery (time slider) tool to travel back in time to extract the images of 2005. Elshayal extracts the images with its associated world files, which aid in registering the images. However, the images (which come in JPEG format) are usually saved in greyscale. Therefore, it was necessary to overwrite the greyscale images from Elshayal with the colour images from GE Pro. This was done by saving it directly from GE Pro-using the “File → Save → Save Image” menu. Subsequently, the images were mosaicked in ArcMap® software.

The process of mosaicking in ArcMap® 10.8 software is described as follows:

i. Add all the GE images using the “Add Data” standard tool
ii. From the standard toolbar, click on ArcToolbox® → Data Management Tools → Raster → Raster Dataset → Mosaic To New Raster
iii. On the “Mosaic To New Raster” window, click on the browse button to add the input rasters (all the GE images) and then select the output location by clicking on the browse button. Next, type the raster dataset name with extension (i.e., GE_Historical_Image_2005.tif) and then set the number of bands to 3 (RGB). Use the default setting for the other parameters and then click OK.

### Ground control point (GCP) survey

GCPs are important to accurately georeference the UAV imagery. Hence, before the UAV survey, 9 GCP targets were evenly distributed across the study site for good coverage. Also, we ensured that the GCPs were placed at least 50 ft (ca. 15 m) from the boundary of the site. This ensures that the control points are free from obstruction and other encumbrances. These GCPs were made of white PVC (polyvinyl chloride) material with a matte finish to prevent glare. The dimensions of the target are 10 cm in width and 1 m in length, in line with standard practice. Next, the GCP targets were measured and coordinated using a differential GPS (Hi-target V30 RTK) in high precision base and rover real-time kinematic (RTK) mode. The base was set up on a temporary control station that was established at approximately 200 m from the first GCP (GCP001), and then the rover was used to measure the crosshair of targets. In differential positioning, the base measures the errors in the GPS signals and then sends corrections to the rover, which in turn helps to enhance the quality of the measured coordinates. Next, the corrected coordinates of the GCP targets were recorded using the Hi-Survey road software installed on the Hi-Target controller and subsequently saved in comma separated value format.

### UAV data

**Fig. 2:**
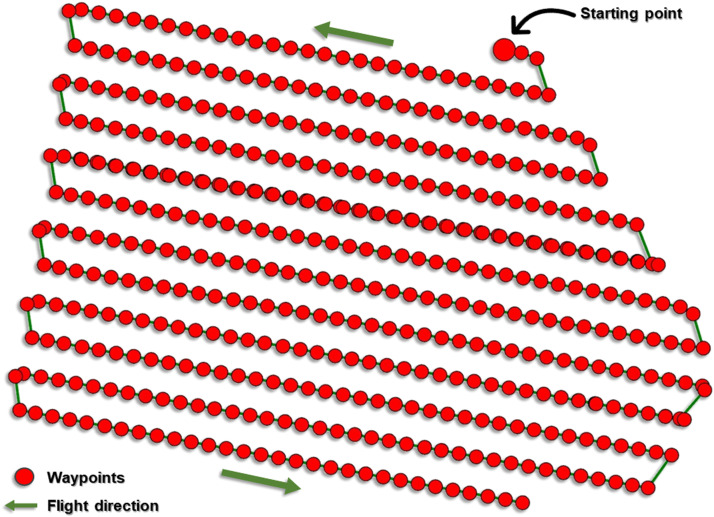
UAV flight path showing the waypoints and flight direction. The green line follows the waypoints in time starting from the large red dot. The visualisations presented in this figure were adapted from Iheaturu et al. [2].

The UAV survey of the study site took place on the 26th of July 2019 using a DJI Mavic 2 Pro-UAV. The DJI Mavic 2 Pro-is equipped with a 1” (2.54 cm) CMOS sensor (20 MP), and it captures images in JPEG format. DroneDeploy® software was used to plan the UAV flight. The DroneDeploy® software allowed us to define the flight path (Fig. 2) and set the flight altitude (61 m), front overlap (60%), side overlap (75%), flight direction (39°), and mapping flight speed (10 m/s) of the mission. The front and side overlap values were recommended by PIX4D [7] for optimal image overlap and quality, while the flight direction was set to the direction of the wind to prevent potential wobbling caused by crosswinds during the flight. The surrounding environment and the scale of the project/area to be mapped must be considered in choosing the flight altitude. The flight mission was scheduled between 12:00 and 13:00 h (GMT +1) local time to ensure the best lighting conditions. This can vary depending on the location. Upon take-off, the DJI Mavic 2 Pro-followed the predefined flight path and captured 470 nadir images in a single flight.

The step-by-step procedure for the UAV flight operation is described as follows:

i. From the take-off and landing point (TOLP), switch on the UAV and its controller and then connect it to the DJI GO 4 application installed on the mobile device.
ii. Calibrate the compass and inertial measurement unit (IMU) following the screen prompts on the DJI GO 4 application. Next, set the home point.
iii. Open the DroneDeploy® application on the mobile device and click on Projects → New Project. Next, click on Create project → type the project name → set the coordinate system (UTM zone 32 N) and click on continue.
iv. Under Maps & Models, select standard and then define the project area. Next, set the flight parameters (e.g., flight altitude, overlap, flight direction, etc.) as described above.
v. Click on “connect drone” to complete the flight checklist. After the flight checklist is complete, click on “Start flight” to begin.

Subsequently, the captured images were imported and processed in Pix4D software to generate an orthomosaic and a digital surface model (DSM).

Once imported into Pix4D, the software automatically geolocated the images using their EXIF (Exchangeable Image File Format) tags, which contain information about the camera model parameters. The EXIF tags are then used to compute the positions of the images. Next, the Output Coordinate System was set to Known Coordinate System → Minna / UTM Zone 32 N, and then the standard 3D Maps processing option was selected. The 3D Maps template is used for generating Orthomosaic, DSM, 3D mesh and point cloud. Subsequently, the processing options were set based on the parameters in Table 1. The processing within Pix4D is done in three main steps: (1) Initial processing, (2) Point cloud and mesh, and (3) DSM, orthomosaic and index. After the initial processing, the GCPs were imported, manually located, and matched on the images using the rayCloud Editor. Next, the camera positions and the internal camera parameters were re-optimised. After steps 2 and 3 were completed, the orthomosaic and DSM generated were saved in “GeoTIFF” format. The SfM-derived orthomosaic and DSM achieved a ground sampling distance (GSD) of 1.43 cm.

**Table 1:** Processing parameters used in Pix4D Mapper.

Processing step	Processing option	Setting
1. Initial Processing	Keypoint image scale	Quarter image size
	Matching image pairs	Aerial grid or corridor
	Targeted number of keypoints	Automatic

2. Point Cloud and Mesh	Image scale	Quarter image size, fast
	Point density	Optimal
	Minimum number of matches	3
	3D textured mesh setting	Medium resolution
	Matching window size	9 × 9 pixels

3. DSM, Orthomosaic and Index	DSM and Orthomosaic Resolution	Automatic
	DSM filters	Check “use noise filtering”
		Check “use surface smoothing”
	Raster DSM	GeoTIFF
	Orthomosaic	GeoTIFF

### Validation

The expected accuracy of UAV surveys is given as 5 - 10 cm (horizontal - X, Y) and 5 - 15 cm (vertical - Z) [2,[8], [9], [10]]. To validate the accuracy of the UAV survey, a point-to-point validation was carried out using the RTK-GPS checkpoints as reference points. All nine (9) ground control points (GCPs) were used for validation, as the use of all GCPs for validation has been shown to improve validation accuracy [2]. More details about the point-to-point validation using all nine (9) GCPs are presented in Iheaturu et al*.* [2]. Summarily, the root mean square errors in the horizontal and vertical positions of the targets (GCPs) were derived as 31.01 mm and 24.34 mm respectively, and this indicates that the results are reliable.

### Feature extraction

To extract the visible features for comparison, it was necessary to register both images (GE historical imagery and orthomosaic) in a common coordinate system to facilitate the comparison. Thus, the GE historical imagery was projected from its original coordinate system (Geographic WGS84) to the coordinate system of the orthomosaic (UTM Zone 32 N). The GE historical imagery was then georeferenced to the orthomosaic in ArcMap 10.8 software using features that were identifiable in both images. This georeferencing helped to eliminate the shifts that resulted from the coordinate transformation. Next, features regarded to be indicators of urban development e.g., building footprints, plots (extrapolated from fences), and roads were digitised for both images and saved as shapefiles. For larger areas, it is recommended to automatically extract these features using automatic and semi-automatic image classification methods such as object-oriented image classification. Subsequently, maps for 2005 and 2019 were produced using the shapefiles created.

Steps for image registration in ArcMap® 10.8 software:

i. From the standard toolbar, click on ArcToolbox® → Data Management Tools → Projections and Transformations → Raster → Project Raster.
ii. On the “Project Raster” window, click on the browse button to select the input raster (i.e., the GE historical imagery in the Geographic WGS84 coordinate system). Next, click on the browse button to set the location folder of the output raster dataset. Next, select the output coordinate system (UTM zone 32 N (Minna)) and click Ok.
iii. Visualise both imageries to identify common features. Next, manually note the coordinates of the features on the orthomosaic and then use those coordinates to georeference the GE historical imagery (georeferencing → Add control points).

Steps for digitisation in ArcMap 10.8 software:

i. Create a working folder on the C Drive.
ii. Create an empty shapefile for the feature: From the standard toolbar click on ArcCatalog® to open it. Next, browse to your working folder and then right-click on it. Then, navigate to New → Shapefile to open the “Create New Shapefile” window. Next, type the name of the shapefile (e.g., Building_footprints), specify the Feature Type (e.g., polygon), and lastly, select the Spatial Reference (UTM zone 32 N (Minna)).
iii. Click on the Editor menu and select Start Editing.
iv. On the “Create Features” window on the right-hand side of the screen, click on a shapefile (e.g., building_footprints) and then start digitising by adding points along the feature's boundary.
v. Repeat steps (ii) to (iv) for all other features such as plots (polygon) and roads (lines).

Steps for map creation in ArcMap® 10.8 software:

i. Symbolise each shapefile as appropriate
ii. Click the View menu → Layout View
iii. Click on File → Page and Print Setup. Next, set the desired map page size and orientation (e.g., A4 size & landscape orientation) and then click Ok.
iv. Click the Insert menu to add elements (e.g., Title, Legend, North Arrow, Scale Bar & Scale Text).
v. Click the View menu → Data Frame Properties → Grid → New Grid → Measured Grid: to create a measured grid.
vi. Set the appearance and intervals as desired and then click Next. Set the axes and labels and click Next → Finish.
vii. Click on File → Export Map and then specify the file name (e.g., GE Map 2005), save as type (e.g., JPEG) and resolution (e.g., 300) in dots per inch, and then click Save.

## Urban development analysis

### Change detection

This study focused on quantifying the changes that occurred in the landscape between 2005 and 2019. To achieve this, we compared the digitized features for both periods. We relied on visual inspection of high-resolution imagery to differentiate between various features. This allowed us to categorize features such as:

• Buildings (completed vs. uncompleted): We classified buildings based on visual cues like the presence of a roof for “completed” and the lack of such feature for “uncompleted”.
• Plots (developed vs. undeveloped): Through visual inspection, we classified plots as “developed” if they exhibited signs of human activity or infrastructure (e.g., presence of buildings, roads, or cultivated land) and “undeveloped” if they appeared to be natural vegetation or bare land.
• Roads (tarred vs. untarred): Visual inspection of the imagery helped distinguish between “tarred” roads with a smooth surface and “untarred” roads with a natural or gravel surface.
• Developed and undeveloped areas: We defined “developed areas” as those exhibiting signs of human modification (buildings, roads, cultivated land) and “undeveloped areas” as those with natural vegetation or bare land.

Following this visual classification, we used metrics like the number of buildings (completed and uncompleted), the number of plots (developed and undeveloped), the length of road (tarred and untarred), and the amount of area developed and undeveloped to facilitate the comparison between 2005 and 2019. The detailed results of this change analysis are discussed in Iheaturu et al. [2].

### Population estimation

The Average Household Size (AHS) dataset was sourced from the Global Data Lab (GDL) database (https://globaldatalab.org/areadata/hhsize/NGA/). The GDL dataset for Nigeria was created by aggregating household survey datasets for 1999, 2003, 2008, 2013, and 2018. Within this database, Abuja's AHS for 2005 was estimated to be 4.26 while the AHS for 2019 was estimated to be 7.25. To obtain the population of the study area, we multiplied the number of residential houses by the AHS (Eq. (1)).

(1) $$P_{tn}=\;NB_{tn}\;\times \;AHS_{tn}$$

Where *P_tn_* = the population of an area for a particular period
*NB_tn_* = number of residential buildings of an area for a particular period
*AHS_tn_* = average household size of the area for a particular period

Since the number of residential buildings for 2005 and 2019 was 185 and 313 respectively, the estimated population for 2005 (P*_i_*) and 2019 (P*_j_*) becomes:

*P_i_* = 185 × 4.26 = 788.1 people
*P_j_* = 313 × 7.25 = 2, 269.25 people

In this study, we assumed that the AHS was the same for every household, although, this may not be the case. Nevertheless, we made this assumption because of the lack of more precise information about the population.

### Urban development intensity (UDI)

The UDI is a measure of the magnitude of development on a site. To have a comprehensive understanding of the UDI of the study site, we utilised four urban development indices (i.e., expansion rate, expansion intensity, average annual expansion index, and urban expansion elasticity index) to quantify the development intensity in the area. The expansion rate *(R),* urban expansion intensity index (UEII), and average annual expansion index (AAEI) were calculated to assess the degree of urban expansion, while the urban expansion elasticity index (EEI) was calculated to ascertain if the development agrees with the rate of population increase. A UEII value of between 0 and 0.28 indicates slow development; 0.28 to 0.59 indicates low-speed development; 0.59 to 1.05 indicates medium-speed development; 1.05 to 1.92 indicates high-speed development; and a value >1.92 indicates very high-speed development [11]. The AAEI values range in the order of 0–5, 5–10, and >10 indicating low-, medium-, and high-speed expansion, respectively [12], while the EEI is reasonable when the value is 1.12. A value lower or higher than 1.12 is considered less reasonable and will likely affect future sustainable urban development [13]. Table 2 shows the description of the urban development intensity indices used in the study.

**Table 2:** Urban development intensity description.

Urban development indices	Formula	Description	Source
Expansion Rate (R)	$R=\frac{U_{j}-U_{i}}{\Delta T}$	The speed of urban development	[4]
Urban Expansion Intensity Index (UEII)	$UEII_{\Delta T}=\left[\frac{U_{j}-U_{i}}{\Delta T}\right]/TLA\times \;100$	The intensity of urban development in the area	[3]
Average Annual Expansion Index (AAEI)	$AAEI=\frac{1}{\Delta T}\times \frac{U_{j}-U_{i}}{U_{i}}\times \;100$	The average rate of urban development in the area	[12]
Urban Expansion Elasticity Index (EEI)	$EEI=\frac{\left(U_{j}-U_{i}\right)/U_{i}}{\left(P_{j}-P_{i}\right)/P_{i}}$	Comparison between the speed of urban development and population	[12]

Where:.

$U_{i}$ and $U_{j}$ are the area of developed plots in periods *i* and *j* respectively.

*i* is 2005 and *j* is 2019.

ΔT is the period of the study (2005 – 2019).

TLA is the total land area.

$P_{I}$ and $P_{J}$ are the number of residents in 2005 and 2019 respectively.

It was found that the Expansion Rate (R) in the area was 1, 333 m^2^/year, the Urban Expansion Intensity Index (UEII) was 0.85, the Average Annual Expansion Index (I) was 1.11, and the Urban Expansion Elasticity Index (EEI) was 0.22 for the period 2005 – 2019.

### Building height assessment

To obtain the heights of the buildings, we first subtracted the digital terrain model from the digital surface model to get the Normalised DSM (nDSM) using the Minus tool in ArcMap® 10.8 (ArcToolbox® → 3D analyst Tools → Raster Math → Minus). Next, the building footprints were converted to points using the Feature to Points tool in ArcMap® 10.8 (ArcToolbox® → Data Management Tools → Features → Feature to Point). Subsequently, the Add Z Information tool (ArcToolbox® → 3D Analyst Tools → 3D Features → Add Z Information) was used to append the heights from the nDSM to the points layer created. Finally, we used the Spatial Join tool (ArcToolbox® → Analysis Tools → Overlay → Spatial Join) to link and join the height attributes of the points to the building footprints. The building heights were then evaluated to ascertain their percentage distribution per height range.

The processes described can be summarised as follows:

i. Obtain the building heights by subtracting the DTM from the DSM, to derive the nDSM
ii. Convert the building footprints from polygons to points
iii. Append the building heights from the nDSM as attributes to the points
iv. Link and join the height attribute to the building footprints
v. Assess the percentage height distribution of the buildings

The heights distribution revealed that 98 (30.6 %) buildings out of the total number of buildings (320) in 2019, both completed and uncompleted, have heights less than 4 m above the ground while 219 (68.4 %) are less than 8 m above the ground. Also, only 3 buildings (1 %) have heights greater than 8 m.

## Limitations

The urban development analysis method described in this study is simplified and can detect and quantify changes in urban and suburban areas and evaluate urban development intensity and parsimonious land use. However, some limitations must be taken into consideration. First, the method focuses on using a combination of spatiotemporal remote sensing and population data from two epochs to assess urban development. While this approach provides valuable insights, incorporating socio-economic data would further enhance the understanding of urban development patterns and dynamics. Socioeconomic factors such as income levels, employment rates, and access to services play a significant role in shaping urban growth and should be considered for a more comprehensive analysis. Second, the method does not assess the level of compliance with land use regulations and the master plan of the area. Evaluating the implementation deficit of these regulations is crucial for understanding the effectiveness of urban planning and management strategies. Future studies should integrate an assessment of regulatory compliance to provide a more complete picture of urban development. Third, the method relies on certain assumptions, such as using a uniform average household size (AHS) to estimate the population. While this approach can be implemented in areas lacking precise population information, more accurate demographic data would yield more precise results in the urban development intensity assessment. Therefore, obtaining and utilizing detailed population data is recommended for improving the accuracy of the analysis. Lastly, the method is applicable for areas of reasonable extent and may not be practical for large-scale urban centers. The use of UAVs to collect high-resolution spatial data is effective for smaller or moderately sized urban areas but becomes impractical for extensive urban regions due to logistical challenges and resource constraints. Applying this method in large cities would require substantial effort, including numerous UAV flights and extensive data processing, making it less feasible. Alternative remote sensing technologies, such as satellite imagery with higher spatial and temporal resolution, might be more suitable for large urban areas.

## Conclusion

This article presents a simplified method that integrates Google Earth historical image and UAV imagery to analyse urban development. It was found that changes in urban developments can be rapidly detected through the comparison of UAV imagery with readily available Google Earth historical imagery. The methods used in this work provide a useful guideline for extracting high-resolution images from Google Earth, conducting UAV surveys, and generating high-quality orthomosaic and DSM. Furthermore, it provides an easy way to estimate the population of an area. This method could support researchers, town planners, and urban authorities in quantifying, analysing, and steering urban development toward parsimonious land use.

## Ethics statements

The authors declare that all ethical practices have been followed in relation to the development, writing, and publication of this article.

## CRediT authorship contribution statement

**Chima Iheaturu:** Conceptualization, Formal analysis, Methodology, Data curation, Visualization, Writing – original draft, Writing – review & editing. **Chukwuma Okolie:** Conceptualization, Methodology, Writing – review & editing. **Emmanuel Ayodele:** Supervision, Writing – review & editing. **Andy Egogo-Stanley:** Methodology, Writing – review & editing. **Solomon Musa:** Methodology, Writing – review & editing. **Chinwe Ifejika Speranza:** Supervision, Writing – review & editing.

## Declaration of competing interest

The authors declare that they have no known competing financial interests or personal relationships that could have appeared to influence the work reported in this paper.

## Data Availability

Data will be made available on request.
